# Optimal treatment and stochastic modeling of heterogeneous tumors

**DOI:** 10.1186/s13062-016-0142-5

**Published:** 2016-08-23

**Authors:** Hamidreza Badri, Kevin Leder

**Affiliations:** Department of Industrial and Systems Engineering, University of Minnesota, Minneapolis, MN 55455 USA

**Keywords:** Tumor heterogeneity, Radiotherapy, Stochastic modeling

## Abstract

In this work we review past articles that have mathematically studied cancer heterogeneity and the impact of this heterogeneity on the structure of optimal therapy. We look at past works on modeling how heterogeneous tumors respond to radiotherapy, and take a particularly close look at how the optimal radiotherapy schedule is modified by the presence of heterogeneity. In addition, we review past works on the study of optimal chemotherapy when dealing with heterogeneous tumors.

**Reviewers**: This article was reviewed by Thomas McDonald, David Axelrod, and Leonid Hanin.

## Background

In recent years there have been many exciting studies that have observed the high levels of diversity present within tumors, (e.g., [42, 74, 82]). In addition to this genomic diversity it is possible for intra-tumor diversity to show up through cell cycle asynchrony or variability in microenviroment. This intra-tumor diversity has the potential to alter the evolutionary trajectory of the tumor cell population under therapy. An important question this raises is how we design optimal treatment strategies when dealing with heterogeneous populations. For example, if we have multiple therapies available there might be tumor subpopulations that respond better to certain therapies. The question then becomes how we optimally administer the various therapies. Addressing this question requires the use of mathematical models to understand the heterogeneity present, and in addition the development of optimization techniques to treat heterogeneous tumors.

In this work, we review past literature that has studied the question of optimal treatment of heterogeneous tumors, as well as stochastic modeling of heterogeneous tumors. The primary focus of the review is on the structure of optimal radiotherapy fractionation schedules when incorporating intra-tumor heterogeneity. A reason for focusing on the radiotherapy setting is that, in simple models of radiotherapy there are well established results for the structure of optimal radiotherapy schedules; see e.g. Badri et al. [5]. Therefore, it is possible to investigate the changes in the optimal schedule as a result of incorporating tumor heterogeneity.

We also review past literature on stochastic modeling and stochastic optimization for the treatment of heterogeneous tumors with chemotherapy or targeted therapy. In this section we look at works that considered stochastic models of heterogeneity in response to therapy, looking at works on both stochastic optimization and stochastic analysis.

## Modeling and optimization in radiotherapy

In this section we will review previous works that studied mathematical modeling and optimization of radiotherapy for heterogeneous tumor cell populations using Linear-Quadratic (LQ) model and its various extensions based on timing effects, cell cycle, hypoxia and cancer stem cell.

### Background on the linear-quadratic model

The LQ equation is widely used to describe the effects of ionizing radiation on normal and neoplastic tissue (For a review see [73]). The basic model states that the fraction of cells that survives a radiation dose of *d* Gy is given by exp ( − *αd* − *βd*^2^) where the radiosensitivity parameters, *α* and *β*, account for non-repairable lesions to DNA and the lethal mis-repair events occurring in the repair process of DNA double strand breaks (DSB), respectively [49, 71]. The initial model has been extended to include the four ‘Rs’ of radiobiology, repopulation of the tumor cells during the treatment period by surviving tumor cells, reoxygenation of hypoxic cells, repair of radiation-induced damage between fractions and redistribution of cells in the cell cycle [96]. These four phenomena are often extended by a fifth ‘R’, which is intrinsic radiosensitivity, defined as the considerable variability between different cell types [85]. These are important determinants of local tumor control after fractionated irradiation, and significantly change the optimal fractionation schemes. In this section, we review several studies that model tumor heterogeneity in radiation fractionation problem and discuss how the conventional optimal fractionation protocols change when considering intra-tumor heterogeneity.

Despite a significant history of predicting doses response curves by the LQ model [13], there is a significant amount of debate as to whether the LQ is appropriate for measuring high dose per fraction effects in stereotactic high-dose radiotherapy (e.g., see [53, 81]). The application of the LQ model is thought to underestimate tumor control at high doses (larger than 10 Gy). Several models have been proposed for improving the prediction of high dose survival curves, e.g. see the models developed by Hanin [51, 52] and Hanin and Zaider [53] or the review by Brown et al. [15] which discusses the validity of LQ model to high dose irradiation of tumors in detail. Since the LQ model is the most widely used model for quantitative predictions of dose/fractionation dependencies in radiotherapy and most models for heterogeneous tumors have been developed based on the same principal structure of the LQ model, we will mainly focus on the LQ model and its extensions in this study.

There are two widely used approaches for delivering radiotherapy: fractionated and continuous radiation. Assuming sufficiently large inter-fraction time, in fractionated radiation, the damage induced in a cell by an acute dose of radiation either causes cell death or complete repair of the cell before the next exposure. Therefore this model leads to memoryless kinetic that can be captured using Markov processes. However this is not the case for continuous irradiation where a longer biological memory of the irradiated cells is stored. See the work of Hanin et al. [57] and experimental studies cited therein for a more detail discussion on how the processes of damage repair/misrepair, cell proliferation and cycling can be modeled by a non-Markovian model. The remainder of this work will largely focus on models of fractionated radiotherapy.

An important problem in radiotherapy is to find the best total treatment size and division of total dose into fractional doses that maximally reduces tumor size while imposing the least amount of damage on surrounding normal tissues (called organ-at-risks or OAR). This problem can be cast as an optimization question and it is commonly referred to as the ‘fractionation problem’. A critical constraint to enforce when locating optimal fractionated schedule is sufficiently low levels of normal tissue toxicity. In order to properly model normal tissue damage, two simultaneous constraints should be imposed: toxicity on early-responding tissue, such as skin and health effects on the late-responding tissue, such as neurons. Usually the concept of biologically equivalent dose (BED), originally motivated by the LQ model, is implemented in clinical practice to measure the biological damage caused by a radiation fractionation scheme in a specified structure. More specifically, the BED for a fractionation regimen with *N* treatment fractions in which radiation dose *d*_*i*_ is administered in fraction *i* (*i* = 1,.., *N*) is given by

$$ BED={\displaystyle {\sum}_{i=1}^N}{d}_i\left(1+\frac{d_i}{\left[\alpha /\beta \right]}\right) $$

where [*α*/*β*] is a tissue-specific radio-sensitivity parameter. The normal tissues toxicity constraints in radiotherapy fractionation problem are mathematically modeled by insisting that BED levels for various OAR stay within prescribed levels [5] or keeping the total number of functional proliferating normal cells more than the required threshold [54]. These constraints can be satisfied by keeping the total dose, fractional dose or dose rate in continuous irradiation within some acceptable levels.

Two possible solutions to the fractionation problem are hyper-fractionated and hypo-fractionated schedules. In hyper-fractionated schedules small fraction sizes are delivered over a long period of time whereas in hypo-fractionated schedules, large fraction sizes are administrated during a short period of radiation delivery. If we maximize tumor control probability (TCP) at the conclusion of treatment, it has been observed that whether hyper or hypo-fractionation is optimal depends on the radio-sensitivity parameters of the normal and cancerous tissue [5, 70, 92]. More specifically if tumor *α*/*β* ratio is smaller than effective *α*/*β* ratio for all normal tissues (defined as (*α*_*i*_/*β*_*i*_)/*γ*_*i*_, where *α*_*i*_/*β*_*i*_ and *γ*_*i*_, denote the radio-sensitivity parameter and sparing factor, respectively, in *i*th OAR), then a single-dosage solution (hypo-fractionated schedule) is optimal, whereas a multiple-dosage solution with equal doses (uniform schedule) is optimal otherwise (hyper-fractionated schedule) [5, 6].

These results are based on the assumption that irradiated cell survival curves are explained by the LQ model, therefore TCP is invariant under rearrangement of fractional doses. However considering more complicated models [55] or different objectives such as minimizing metastatic risk [7, 8] instead of maximizing TCP may result in the optimality of non-standard fractional schedules. These schedules are formed using the front loading principle: administering the maximum possible dose as soon as possible. Moreover, as a result of these emerging alternative models and objectives, other factors such as the time point at which the performance criteria is evaluated may play an important role in the structure of optimal schedules, e.g. see Zaider and Hanin [102] and Badri et al. [7].

### Intra-tumor heterogeneity

The uncertainties in radiotherapy treatment can be categorized into two groups: inter-patient variability and intra-tumor heterogeneity. Inter-patient variability stems from heterogeneity in patient-specific variables such as the sensitivity of their normal tissues and tumor to radiation (*α*/*β* ratio), the growth rate of their tumor or the healing kinetics of normal tissues. Several studies addressed these uncertainties using different techniques. Badri et al. [6] proposed a stochastic optimization formulation to incorporate inter-patient variability in tumor and normal tissue radiosensitivity parameters (*α* and *β*) and sparing factor of the OAR into the scheduling optimization problem. Hanin and Zaider [54] developed a mechanistic approach that models post-irradiation normal tissue toxicity when considering inter-patient variation of kinetic parameters. On the other hand, to improve the efficacy of radiation therapy, it is necessary to study the role of intra-tumor heterogeneity, since it significantly changes the tumor response curves [34, 58]. The range of cell sensitivity comes from inherent genetic and epigenetic differences among the tumor cells and from temporal variations arising from the asynchronous cell cycle phases and variable micro environmental conditions during therapy. The focus of the present work is to review studies that model intra-tumor heterogeneity and present where possible novel optimization problems that arise from these models.

In [56], Hanin et al. studied the role of radiosensitivity variation amongst cancer cells on optimal radiotherapy fractionation schemes. They used a new criterion developed by Rachev and Yakovlev that considers the difference between weighted survival probabilities for normal and neoplastic cells, where tumor cell radiosensitivity is considered as a random variable with a known distribution function [76]. For several special cases, the exact solution of optimal fractionation is obtained and an iterative approximation methodology is designed when it is not possible to compute the exact optimal fractionation schedule.

**Fig. 1:**
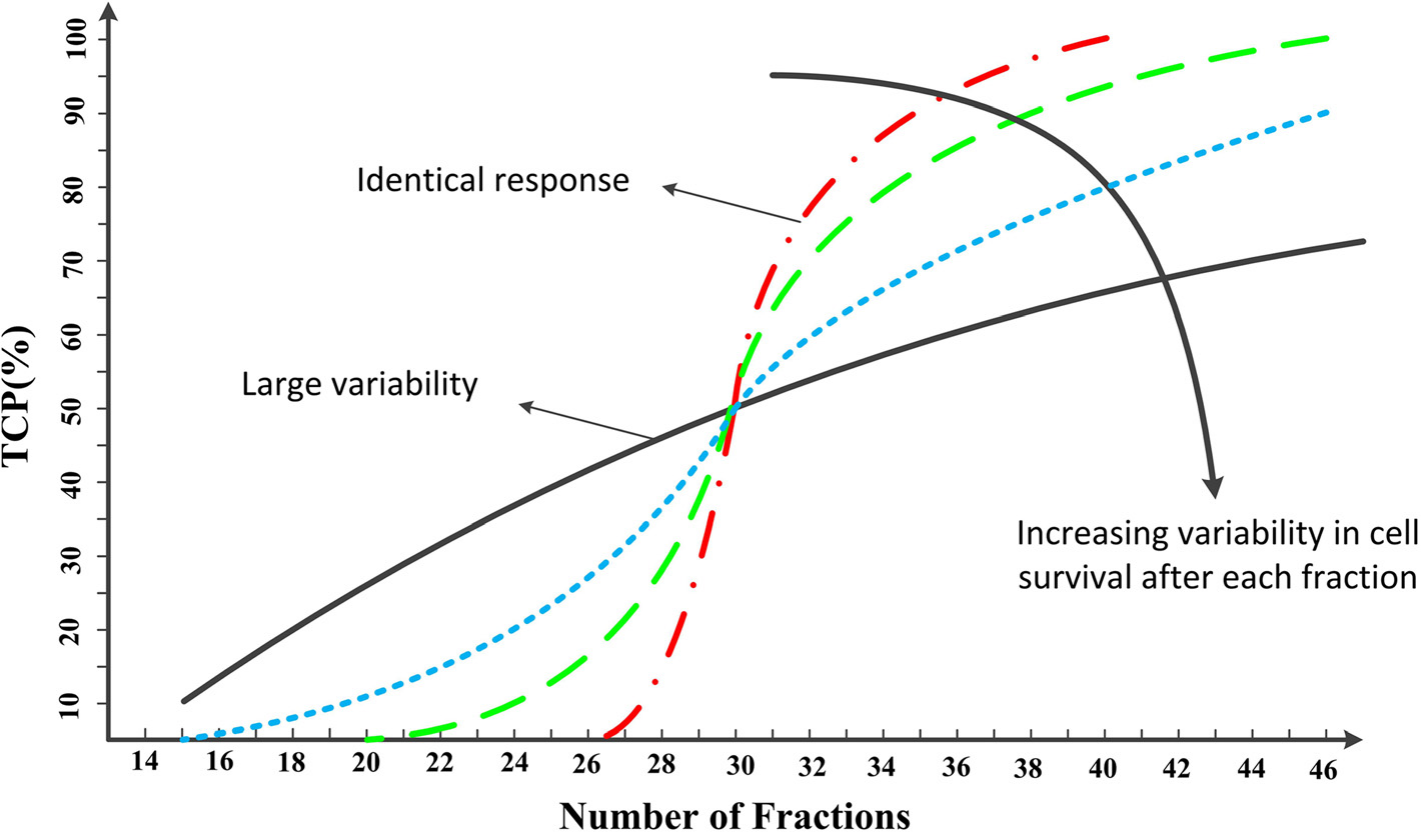
Relationship between TCP and number of 2.0 Gy fractions for different tumor population variabilities based on the model developed by Zagras et al. [101]. The fraction of surviving cells is assumed to be normally distributed. The standard deviation of the normal distribution measures the homogeneity of tumor cells

Several studies have suggested that intra-tumor heterogeneity accounts for variability observed in radiobiological parameters and TCP versus dose [58]. Zagars et al. categorized the cells existing in a tumor into three main subclasses: the radio-sensitive cells which are controllable with radiotherapy, the radio-resistant cells that are not susceptible to damage from therapeutic radiation, and the stochastic fraction, which includes those cells with tumor control probability between 1 and 99 % [101]. The population TCP over total delivered dose curve, so-called TCP/D, can be modeled as a weighted summation of individual TCP/D curves, where the weights are estimated based on the relative frequency of the different types of tumor cells in the population. It was observed (see Fig. 1) that intra-tumor heterogeneity flattens the tumor dose–response curves [90, 101].

### Timing and 4R’s effects on tumor heterogeneity

The fraction of surviving cells after a dose of radiation not only depends on dose and tumor radio-sensitivity parameters, but also it typically depends on the time-course of dose delivery [88]. Timing affects cell killing due to several reasons such as DNA repair and misrepair, tumor repopulation, redistribution and reoxygenation [48, 49]. The basic LQ model typically assumes that tumor radio-sensitivity parameters (*α* and *β*) and repopulation are constant over the time course of radiotherapy. This implies the failure of the simple version of the LQ equation exp ( − *αd* − *βd*^2^) to capture the dynamics of reoxygenation and repopulation throughout the course of treatment. Mathematical models for ionizing radiation therapy, applied to multicellular populations whose cells have time-dependent radio sensitivity have been studied widely [17, 60]. However in some cases such as heterogeneity associated with cell sensitivity and proliferation rate when fractionated irradiation with sufficiently many fractions or protracted continuous radiation is implemented, it is possible to only consider the homogeneous subpopulations of the most resistant and/or fastest growing cells. This is due to the fact that usually slowly growing tumor cells and sensitive subpopulations die out after commencement of therapy, and therefore it is sufficient to design the therapy to target the fast growing tumor cells and resistant population. As an example see the mathematical model developed by [54] to model the number of proliferating as well as non-proliferating normal cells as a function of time post treatment when incorporating the selection of the fastest growing subpopulation to capture the tissue damage at the conclusion of therapy and of the subsequent healing kinetics.

**Fig. 2:**
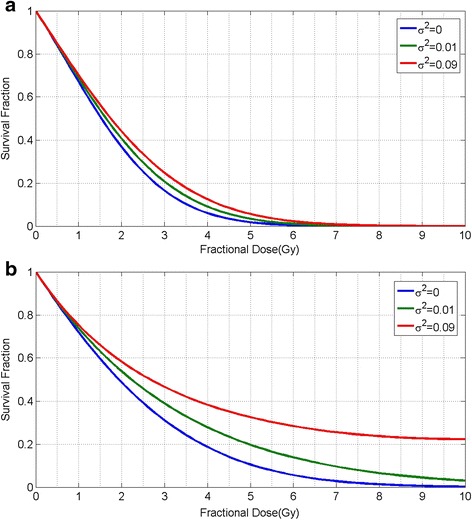
Cell survival curves illustrating the effect of tumor heterogeneity on surviving fraction of cells after a single dose of radiation based on Eq. (3) **a**) This plot is shown for *α* = 0.3 and *β* = 0.1 **b**) This plot is shown for *α* = 0.3 and *β* = 0.03

Hlatky et al. [60] studied the variable response of tumor cells to therapeutic treatment in ionizing radiation by modeling the resensitization process; which includes redistribution and reoxygenation. The resensitization process states that after the dose is delivered, a large fraction of damage occurs among the radiosensitive cells, resulting in decreased average radiosensitivity. However these changes are reversible; and the remaining subpopulation are driven into more radiosensitive states as time passes [14, 60]. Considering a smooth function for absolute number of cell that have sensitivity *α* at time *t*, i.e. *n*(*α*, *t*), we can write the equation explaining the fluctuating diversity of a population with fixed size using a Kolmogorov forward equation as (see [60] for more details)

1 $$ \frac{\partial n\left(\alpha, t\right)}{\partial t}=-\left(\alpha \overset{.}{D}-\frac{1}{2}\kappa {u}^2\right)n+k\frac{\partial }{\partial \alpha}\left(\left(\alpha -{\alpha}_0\right)n+{\sigma}^2\frac{\partial n}{\partial \alpha}\right) $$

where $\overset{.}{D}$ is the dose rate, *u* denotes the average number of DSB per cell, $\frac{1}{2}\kappa\ {u}^2$ shows the average rate at which binary misreapirs removes DSB by lethal rearrangements, *k* displays the rate at which cells change their radiation sensitivity, and *α*_0_ and *σ*^2^ represent the mean and variance of random variable *α*, respectively. Note that in the case of a homogeneous tumor, *σ* = 0, Eq. (1) becomes the deterministic model developed by Sachs et al. in [80] which adds the enzymatic modification of the immediate damage through a Markov process to the basic LQ model. Considering tumor population in the long term, it was shown that the solution to the Eq. (1) gives the surprising simple result of

2 $$ N\left(\infty \right)=N(0) \exp \left(-{\alpha}_0\ D+\left(\frac{1}{2}{\sigma}^2G(kT)-\beta G\left(\lambda T\right)\right){D}^2\ \right) $$

where *N*(*t*) shows the total population at time *t*, *D* is total radiation dose delivered for period (0,T), and *G* is the Lea-Catcheside function [60]. Equation (2) can be considered as the elementary LQ model with *α* being replaced by its average *α*_0_, and *β* being replaced by its modified value. Results of their analysis support the hypothesis that the therapeutic paradigm of low dose rate or fractionated radiation can help conquer radioresistance in hypoxic tumors [91, 97]. This is due to the fact that a large fractionation interval (parameter *T* in (2)) allows the tumor population to complete the reoxygenation process and thereby the tumor population radio-resistance due to oxygenation status will be minimized. This phenomenon is supported by a smaller coefficient for *D*^2^ in Eq. (2). One year later, Brenner et al. developed a parsimonious model to include the resensitization effect into the LQ model. In the extended model, designated LQR, survival is written as a function of dose *d* as

3 $$ \exp \left(-\alpha d-\left(\beta -\frac{1}{2}{\sigma}^2\right){d}^2\right) $$

where the term $\frac{1}{2}{\sigma}^2{d}^2$ refers to cellular diversity, and is given by the uncertainty about the cell kill by one-track action of radiation, i.e. parameter *α* [14]. The cell survival values based on Brenner et al. model (Eq. (3)) are plotted in Fig. 2 for values of *σ*^2^ = 0, 0.01 and 0.09 for cell population without, low and high diversity, respectively. By comparison of cellular diversity effect for tumors with different values of $\frac{\alpha }{\beta },$ we observe a more significant effect for tumors with large values of $\frac{\alpha }{\beta }$, e.g. 10 for prostate cancer (Fig. 2b), compared to tumors with a small value of $\frac{\alpha }{\beta }$, e.g. 3 for head and neck cancer (Fig. 2a).

Optimization of radiotherapy treatment within the Hlatky model which includes time dependence of sublethal damage repair has been studied by Yang and Xing in [99]. It has been observed that incorporating these effects into the LQ model may give rise to optimal non-uniform fractionation schedules where fractional doses at the beginning and end of each irradiated week become significantly greater than others. Furthermore it was observed that the hyper-fractionation schedule gives an insignificant advantage over hypo-fractionation or a standard regimen.

### Cell cycle

Another reason that radiotherapy cell killing depends on timing, not just total dose, is the process of the mitotic cycle. Tumor cells respond differently to radiation in different cell phases of the cell cycle [11], e.g. cells within the *G*_0_-phase of the cell cycle, quiescent cells, possess a lower level of radio-sensitivity than proliferating cells that are in the *G*_1_, *S*, *G*_2_, *M*-phases [23, 73]. Therefore for tumors with asynchronous cells, increasing radiation delivery time,*T*, increases tumor radiosensitivity. This makes sense because at first the radiation kills the cells in more sensitive phases, and then radioresistant cells, e.g. those are in *G*_0_-phase, have time to reach more sensitive phases. Also due to cell arrest in the most sensitive phases of cell cycle, protracted radiation promotes synchronization. Chen et al. studied the effect of cell cycle redistribution on the population resensitization when ignoring the quadratic misrepair of radiation damage, *β* [17]. They used a Mc-Kendrick-von Foerster equation adjusted for the first track radiation cell kill to model the age dependent cell dynamics as

4 $$ \frac{\partial n\left(a,t\right)}{\partial t}=-\frac{\partial n\left(a,t\right)}{\partial a}-\overset{.}{D}\alpha (a)n\left(a,t\right)-g(a)n\left(a,t\right) $$

where *n*(*a*, *t*)*da* shows the density number of cells in the age range (*a*, *a* + *da*) at time *t* and *α*(*a*) shows the tumor radiosensitivity at age *a*. They observed that the tumor population resensitization effect occurs as the duration *T* of irradiation is increased from essentially zero times to short, and sufficiently small finite times. They concluded that population resensitization is proportional to *T*^2^ and $\exp\ \left(-\alpha (a)D\right)\ {\left(\frac{Dd\alpha }{da}\right)}^2$ and the resensitization happens when *T* is small and the cell population is in a stable age-distribution phase before irradiation, which in this case happens regardless of how the radiation cell kill, function *α*(*a*), depends on age. Hahnfeldt and Hlatky generalized the model proposed by Chen et al. beyond constant-dose-rate irradiation and small *T* in more explicit terms [48]. They have used the same equation described in (4) and have shown mathematically that variation with the time of resensitisation due to redistribution is not monotonic but damped oscillatory. They found that spreading a dose of *d* Gy over a longer period of time in any way is more desirable and results in higher TCP than delivering an acute dose of equal magnitude. They proved that this result continues to apply regardless of age-dependent sensitivity and mitosis rate functions chosen.

In [23], Dawson and Hillen have considered extensions to the TCP model developed by Zaider and Minerbo [103] to include the quiescent states and cell cycle dynamics. The model is based on a birth-death process and generalizes the Zaider and Minerbo TCP formula, aiming to include cell cycle effects according to the idea that assumes the cell populations split into two compartments which represent an active phase (*G*_1_, *S*, *G*_2_, *M*) and a quiescent phase(*G*_0_). If the clonogenic cells do not enter a *G*_0_ phase, which is modeled with considering the transition between both compartments during radiotherapy, then the model equally applies for a splitting into *S*, *G*_2_, *M* and *G*_1_ phases. The key assumption is that actively proliferating cancer cells are much more susceptible to radiation damage than quiescent cells. The basic model states that the expected number of cells in active,*N*_*A*_, and quiescent compartments, *N*_*Q*_, satisfy a system of differential equations as

5 $$ \frac{\partial {N}_A}{\partial t}=-\mu {N}_A+\nu {N}_Q-{\lambda}_A(t){N}_A-{h}_A(t){N}_A,\ \frac{\partial {N}_Q}{\partial t}=2\mu {N}_A-\nu {N}_Q-{\lambda}_Q(t){N}_Q-{h}_Q(t){N}_Q $$

where *μ* is the rate of active cell division, *ν* describes the transition from quiescent compartment into the cell cycle, *λ*(*t*) shows the death rate of different types of cells at time *t* and *h*(*t*) explains the radiation induced death rate in different compartments. Note that since active cells are more radiosensitive, we have*h*_*A*_(*t*) > *h*_*Q*_(*t*). The original model of Dawson and Hillen have been taken and extended to describe more complex systems or models with more compartments [25, 32, 59, 68]. Analysis of Dawson and Hillen active-quiescent radiation model and its comparison to LQ model confirms that a larger *α*/*β* ratio relates to a fast cell cycle and indicates the presence of a significant quiescent compartment, while a smaller ratio is associated with a slow cell cycle [23]. These comparisons were performed under the LQ model assumptions which allowed the authors to construct a relationship between proliferation and transition rates, *μ* and *ν* in Eq. (5), respectively, in their model with *α* and *β* parameters in the LQ model. Therefore we can conclude that for the tumor population with a substantial quiescent compartment, which indicates a large value for *α*/*β* ratio, hyper-fractionated schedules provide a better TCP than the hypo-fractionated schedules (see [70] or Badri et al. [5]). These types of analysis are indeed the future direction of the cell cycle modeling in TCP, i.e. the inclusion of cell cycle and diversity of the cellular radiosensitivity of a tumor in optimization of radiation dosing schedules.

### Hypoxia

**Fig. 3:**
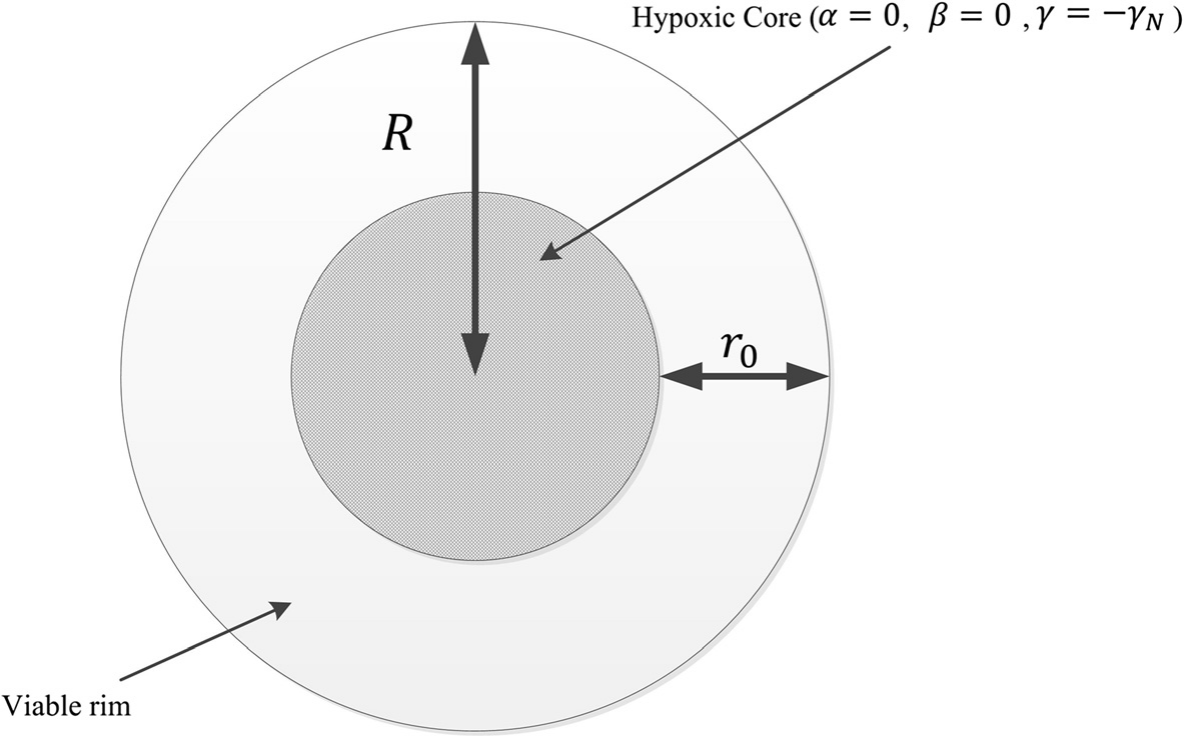
Tumor geometry in the mathematical model by [94]. Tumor cells are insensitive to radiation at hypoxic core and die at rate *γ*_*N*_ per day

Hypoxia plays a significant role in the reduced response to radiation [45, 78]. Specifically, a cell in the tumor may experience changes in radiosensitivity due to a change in the tumor microenvironment, e.g., a decrease in oxygen levels to a hypoxic state. As a tumor shrinks and a significant proportion of cells are killed, the radius of the tumor cord shrinks; diffusion-limited hypoxia decreases and necrotic or hypoxic regions become smaller and may finally disappear. Consequently there is no nutritional deprivation leading to cell death. Therefore the net repopulation rate increases as the tumor shrinks [40]. This idea has been utilized to incorporate the volume-dependent sensitivity and repopulation effect in the LQ model [12, 16, 79, 94]. Several experiments provide evidence that indicates that radio-sensitivity and growth rate in tumor spheroids decrease as the distance from the nutrient supply increases [21, 87, 89]. Hence a simple way to model this phenomena is assuming that tumor cell sensitivity to radiation, *α* and*β*, and the tumor net repopulation rate,*γ*, depend upon the cell radial distance, *r*, from the center of the tumor, and on the current tumor radius, *R* [94]. Then if we assume all of these parameters take on a fixed well-oxygenated level at the tumor surface (i.e. *α* = *α*_0_, *β* = *β*_0_ and *γ* = *γ*_0_ at *r* = *R*) and decrease linearly as *r* decreases, we can compute the radio-sensitivity parameters and tumor growth rate as a function of *r* ∊ [0, *R*] for *R* < *r*_0_ as (see Fig. 3 and [94] for more details)

6 $$ \alpha \left(r,R\right)={\alpha}_0-\frac{\alpha_0}{r_0}\left(R-r\right),\ \beta \left(r,R\right)={\beta}_0-\frac{\beta_0}{r_0}\left(R-r\right),\ \gamma \left(r,R\right)={\gamma}_0-\frac{\gamma_0}{r_0}\left(R-r\right) $$

and for *r* ∊ (*R* − *r*_0_, *R*] and *R* > *r*_0_ as

7 $$ \alpha \left(r,R\right)=\frac{\alpha_0}{r_0}\left(r-R+{r}_0\right),\kern0.5em \beta \left(r,R\right)=\frac{\beta_0}{r_0}\left(r-R+{r}_0\right),\ \gamma \left(r,R\right)={\gamma}_0 - \frac{\gamma_0}{r_0}\left(r-R+{r}_0\right) $$

As discussed by the authors, the linearity assumptions in Eqs. (6) and (7) may not be compatible with the physics of oxygen diffusion and were chosen for their parsimony and computational feasibility. The actual situation in vitro and in vivo is significantly more complex, e.g. the oxygen enhancement ratio depends on the fraction size [49], therefore a more complicated model is required to explain the tumor radiosensitivity as a function of radial location. Also Eqs. (6) and (7) are based on the assumptions that the net rate of spontaneous cell death decreases as the tumor shrinks, which is applicable for most types of tumors (e.g., well-differentiated squamous cell cancers) and is consistent with experimental results [87, 89].

If we show the tumor radius and number of tumor cells at time *t* by *R*_*t*_ and *n*_*t*_, respectively, and we assume the density of cells per unit volume in the spherical tumor to be *θ*, then we have ${n}_t=\frac{4}{3}\theta \pi\ {R}_t^3$. Using LQ formulation adjusted for exponential tumor growth [49], the expected change in number of tumor cells after a dose of size *d* is (see [94] for more details)

8 $$ {\overset{.}{n}}_t={n}_t\left[\gamma \left({R}_t\right)-\alpha \left({R}_t\right){d}_t-2\sqrt{\beta \left({R}_t\right)}{d}_t{\displaystyle \underset{0}{\overset{t}{\int }}}\sqrt{\beta \left({R}_t\right)}\ {d}_s{e}^{-\mu \left(t-s\right)}ds\right] $$

where *μ* is tumor repair rate. Substituting Eqs. (6) and (7) and using equation ${\overset{.}{n}}_t=4\theta \pi\ {R}_t^2\ {\overset{.}{R}}_t$, we can write Eq. (8) in terms of *Ṙ*_*t*_ and *R*_*t*_ and forms the basis of the optimal control problem. Wein et al. proposes a dynamic programming approach to numerically solve this problem. The resulting optimal protocols suggest a non-standard time varying schedules with irregular time intervals between fractions, administering larger fractions before longer breaks, such as afternoon sessions or Fridays, and shorter fractions before shorter breaks, such as morning sessions [94]. Wein et al. proposed two main reasons for this phenomenon. First the large fractions make up for tumor repopulation during overnight or weekend breaks. Second the tumor size is smaller at the end of the week, i.e. Fridays, and smaller tumors are more sensitive to radiation. They also observed that as the tumor shrinks during therapy, it is optimal to increase the doses on Friday afternoons. Based on their model, as the tumor shrinks, *α*(*R*)/*β*(*R*) becomes smaller which leads to the optimality of hypo-fractionated schedules.

### Cancer stem cell

The existence of cellular heterogeneity in solid tumors may originate from a number of sources, including hypoxia, cell cycle asynchrony, infiltration of normal cells, vascular structures and stroma into the tumor and the hierarchical structure of the cell populations from which cancers arise. The cancer stem cell (CSC) model of tumorigenesis has received significant attention in recent years. CSC refers to a subset of tumor cells that has the ability to self-renew and generate differentiated progeny which make up the bulk of a tumor [77]. Existence of CSCs has been identified in different cancers such as acute myeloid leukemia [26] breast cancer [1] and brain tumors [84]. The definition of CSC implies that an anticancer therapy can control a tumor, i.e. permanent local tumor control, only if all CSCs are eradicated. Therefore it is possible that removal of CSCs is the crucial determinant in curing cancer and eradicating tumor cells [10].

The concept of CSCs has profound clinical implications. In particular, CSCs in solid tumors are more resistant to anti-cancer treatments, such as radiotherapy [9, 50, 75, 98]. Mathematical modeling that integrates this complexity has been used to analyze and predict the evolutionary dynamics of heterogeneous tumor populations caused by the hierarchical natures of the cell populations. A dual-compartment linear-quadratic model (DLQ) is usually implemented to study tumor hierarchical intrinsic heterogeneity [67, 93]. DLQ assumes there exist two cell populations in a solid tumor, CSCs and differentiated cancer cells (DCC), where CSCs form the minor subpopulation of a solid tumor. CSCs are able to produce more CSCs as well as DCCs and are described as the more radio-resistant subpopulation (have lower values of *α* and *β*). The radiation response model is constructed as

9 $$ S(d)=F\times \exp \left(-{\alpha}_sd-{\beta}_s{d}^2\right)+\left(1-F\right)\times \exp \left(-{\alpha}_dd-{\beta}_d{d}^2\right) $$

where *S*(*d*) represents the fraction of surviving cells after delivering an acute dose of radiation, *F* represents the fraction of CSCs out of all cells, and (*α*_*s*_, *β*_*s*_) and (*α*_*d*_, *β*_*d*_) show the radiosensitivity parameters in CSC and DCC, respectively. The interplay between CSCs and DCCs can be modeled by using the ODE introduced in Hillen et al. [59] as (10)

10 $$ \begin{array}{c}\hfill \frac{\partial {N}_s}{\partial t}=\left(2p-1\right){\mu}_sk\left(N(t)\right){N}_s(t)\hfill \\ {}\hfill \frac{\partial {N}_d}{\partial t} = 2\left(1-p\right){\mu}_sk\left(N(t)\right){N}_s(t)+{\mu}_dk\left(N(t)\right){N}_d(t)-{a}_v{N}_d(t)\hfill \end{array} $$

where *N*_*s*_(*t*) and *N*_*d*_(*t*) are the volume fractions of CSCs and DCCs, respectively. The function *N*(*t*) is the total volume of tumor normalized between 0 and 1 which is equal to *N*_*s*_(*t*) + *N*_*d*_(*t*), *p* is the probability of symmetric CSC division, and *μ*_*s*_, *μ*_*d*_and *a*_*v*_ define the CSC growth, DCC growth and DCC apoptosis rate, respectively. *k*(*N*(*t*)) is a constraint defined as max {1 − *N*(*t*)^*σ*^, 0} for a *σ* ≥ 1 and keeps the total volume fraction less than 1. In [4], Bachman and Hillen used the ODE Eqs. in (10) and showed that the differentiation therapy proposed by Youssefpour et al. [100], which is defined as the combination of radiotherapy and chemotherapy where the chemotherapeutic agent pushes CSCs into the differentiation stage, can have large beneficial effects in head and neck cancer, brain cancers and breast cancer for the patient increasing treatment success and reducing side effects.

**Fig. 4:**
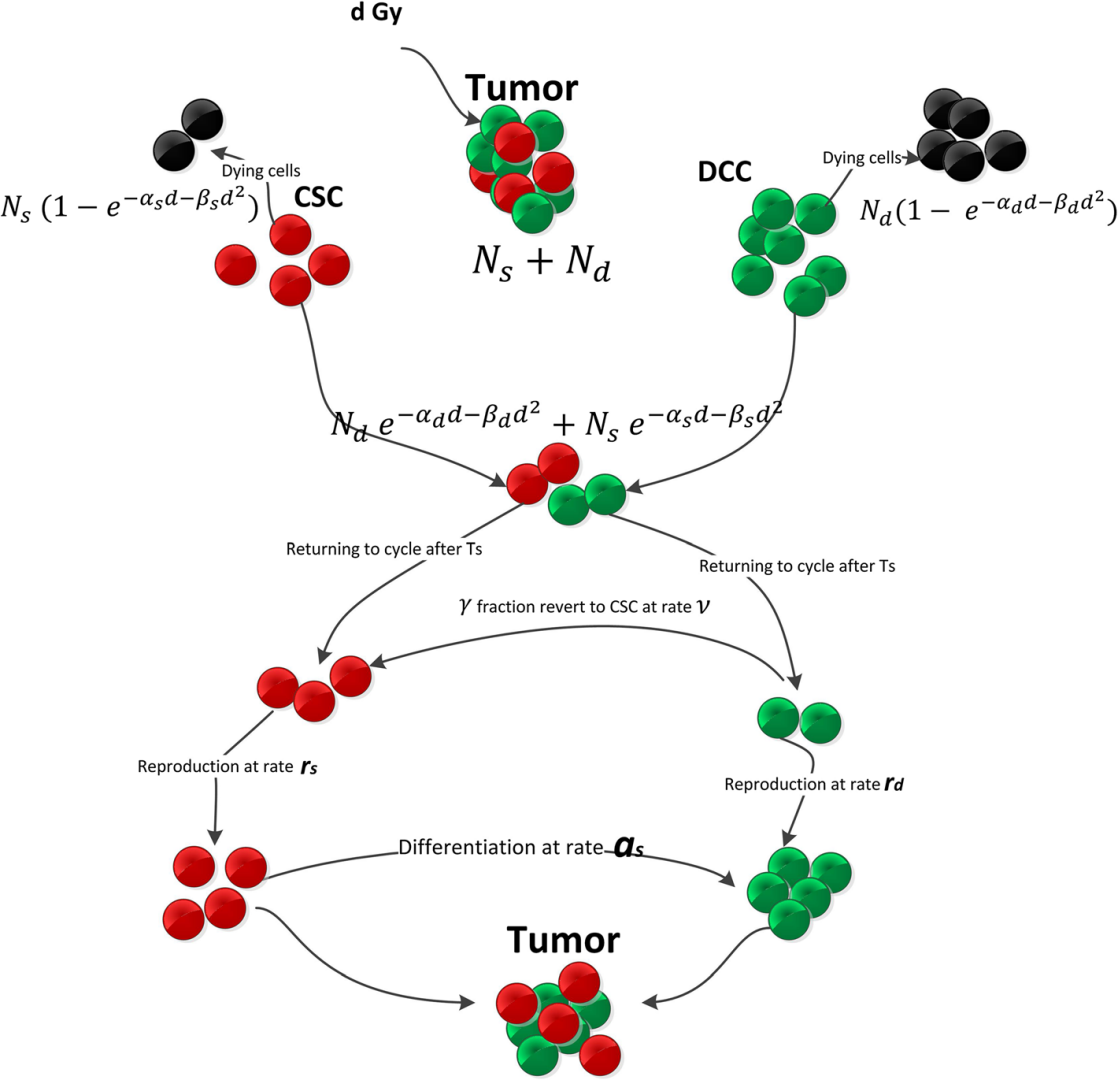
Mathematical model described in [67]

Leder et al. developed a model to study the reversible phenotypic interconversions between the CSCs and the DCCs in glioblastomas (GBM), i.e. radiation may induce DCCs to dedifferentiate into CSCs [67]. They assumed that the increased radiosensitivity of DSCs to be expressed in relation to the CSCs radioresistance, measured by the parameter *ρ* ∊ (0, 1], i.e. *α*_*s*_ = *ρα*_*d*_ and *β*_*s*_ = *ρβ*_*d*_. This simplifying assumption enabled the authors to characterize the sensitivity of CSCs to radiation by a single parameter, *ρ*. The model is described in Fig. 4. The model stipulates that *t* hours after the previous dose of radiation, the fraction of DCCs capable of reversion to CSCs is given by $\gamma (t)={\gamma}_0{e}^{-{\left(t-{a}_0\right)}^2/{a}_1^2}$ (note that *γ*(*t*) = *γ*_0_ for the first dose of radiation), for some constants *γ*_0_, *a*_0_ and *a*_1_ and the fraction of surviving cells can be computed based on the LQ model. They predicted several optimal radiation strategies that substantially enhanced survival in experimental studies using a mouse model of glioblastoma. The resulting optimized schedules recommend a non-uniform schedule delivering larger fractions at the beginning and toward the end of the therapy. In a follow up work, Badri et al. used the Leder model to consider fractionated schedules that have optimal survival while, maintaining acceptable levels of toxicity in early- and late-responding tissues [5]. They derived the closed form solution to the problem and proved that the optimization problem can be split into two separate optimization tasks that can be tackled independently. The first model involves optimization of dose per fraction and the optimal total dose, and the second model optimizes inter-fraction intervals between radiation doses. It was observed that normal tissues sparing factors and radiosensitivities, and the magnitude of the *α*/*β* ratio for tumor are determinant factors defining the optimal radiation scheme, i.e. for low (high) values of tumor *α*/*β* ratio, the hypo-fractionated (hyper-fractionated) schedule is optimal. For the time-dependent model, the optimal inter-fraction intervals only depend on the time dynamics of the dedifferentiation process and treatment duration. In particular it was observed that optimal inter-fraction intervals are equal to the dose spacing that leads to the maximal amount of cell reversion to the stem-like state, i.e. *a*_0_.

Several stochastic and cellular automata models have been used in more complicated simulation based studies of complete tumor cell kinetics during radiation therapy. Gao et al. used an integrated experimental and cellular Potts model to simulate glioblastoma population growth and response to irradiation [41]. They found that in order to maintain the tumor population following radiotherapy, surviving glioma CSCs in vitro increase their rate of self-renewal, i.e. the fraction of CSCs in the populations is increased after radiation. By comparing acute and fractionated irradiation response, the authors observed that the relative increase in fraction of CSCs in tumor population after fractionated treatment cannot be explained merely by radioresistance of CSCs. This simulation based model suggests that repeated exposure to radiation might increase the symmetric division rate of CSCs, which eventually may lead to accelerated repopulation of CSCs. A series of in vivo 4D simulation models for GBM explore the tumor growth dynamics and response to radiation, considering vasculature, oxygen supply and radiosensitivity [2, 27, 28]. These works clustered cells into dynamic classes based on the mean cell cycle phase durations over the various cell cycle phases and used a linear quadratic model to describe the number of cells killed. They associated p53 mutations with increased radioresistance and inefficient clinical outcome for patients with GBM, as suggested by Haas-Kogan et al. [46]. Evaluating the response to treatment for different fractionation regimens revealed that hyper-fractionated schedules may lead to an improvement in local tumor control compared to standard schedules.

## Chemotherapy

In this section we will review previous works that studied stochastic modeling and optimization of chemotherapy for heterogeneous tumor cell populations.

### Optimization models

There is a vast literature on the mathematical modeling and optimization of the delivery of chemotherapy, e.g., see the three review papers Shi et al. [83], Swan [86] and Kimmel and Swiernak [64], or the textbook by Martin and Teo [69]. In this large literature optimization problems are formulated to optimally achieve a desired patient outcome subject to various constraints. Several works in these reviews follow an optimal control approach, e.g., Swan mentions several problems of this form [86]. Specifically, these works assume that cancer cell population satisfies a differential equation that depends on the drug concentration, e.g.,

$$ \frac{\partial x}{\partial t}=x\left[f(x)-h(u)\right], $$

where *x* is the cancer cell population size and *u* is the drug concentration level. Also *f* and *g*are arbitrary functions that represent density dependence and drug induced cell kill respectively. Then a cost function is specified, e.g.

$$ J\left(x,u\right)={\displaystyle \underset{0}{\overset{t}{\int }}}\left[\omega \left(x(t)\right)+\rho\ u{(t)}^2\right]dt, $$

and the goal is then to use optimal control methodology to numerically identify the optimal drug concentration profile *u*. There is a wide range of works on models of this kind and we refer the reader to the reviews Shi et al. [83], Swan [86] and Kimmel and Swiernak [64] for further examples.

Given the large amount of literature on this topic, we focus in the remainder of this section on works related to optimization of stochastic models of the treatment process for heterogeneous tumors with resistant subtypes.

### Optimization of stochastic models

**Fig. 5:**
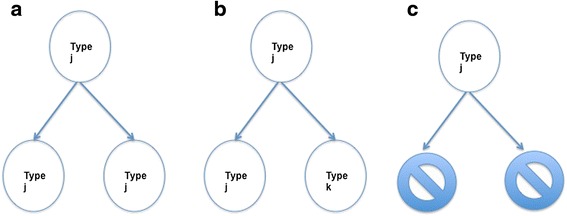
In panel (**a**), we show an event where a type-j replicates without mutation, panel (**b**) a type-j has a single mutated offspring a type-k cell, and in panel (**c**) a type-j cell dies

The majority of stochastic models of tumor response to chemotherapy have been based on the continuous time binary multi-type branching process framework (see e.g. [3]). In this modeling framework there are *m* possible cell types, and all cells of a given type behave in a statistically identical fashion independently of all other cells present. In particular a cell of type-*i* well wait an exponentially distributed amount of time with mean 1/*a*_*i*_ before a birth/death/mutation event. During this event the type-*i* cell produces offspring of type (*j*_1_, …, *j*_*m*_) with probability *p*^(*i*)^(*j*_1_, …, *j*_*m*_), where *j*_1_ + … + *j*_*m*_ ∊ {0, 2} (see Fig. 5). The multi-type branching process is specified by the vector $\overrightarrow{a} = \left[{a}_1\cdots\ {a}_m\right]$ and the vector valued mapping

$$ P\left({j}_1,..,{j}_m\right)=\left(\begin{array}{c}\hfill {p}^{(1)}\left({j}_1,\dots, {j}_m\right)\hfill \\ {}\hfill \vdots \hfill \\ {}\hfill {p}^{(m)}\left({j}_1,\dots, {j}_m\right)\hfill \end{array}\right) $$

The long term behavior of a multi-type branching process is easily deduced from this information. In particular, one forms a mean matrix *M* = (*m*_*ij*_), where *m*_*ij*_ is the expected number of type-*j* offspring a type-*i* offspring will produce. If the maximum eigenvalue of the matrix *M* is less than or equal to one then the branching process is guaranteed to go extinct, while if it is greater than 1 then the branching process can either go extinct or its size diverge to positive infinity. Therefore understanding the long term behavior of the branching process is straightforward. When studying the problem of drug resistance in cancer one is often interested in the behavior of the process over a long (but finite) time interval, and therefore it is not sufficient to simply look at the maximal eigenvalue of *M*. For an example of other techniques that can be used see e.g. Durrett and Moseley [30], Iwasa et al. [61], Haeno et al. [47], or Durrett et al. [31].

When modeling drug resistance in chemotherapy, a standard approach would be to assume that initially most cells are type-1, which is assumed to be sensitive to some first line therapy. Thus during treatment with this first line therapy the type-1 cells will begin to decrease; however, these cells may mutate to a different type of cell that can grow under the first line therapy. This type of cell may decline under a second line therapy; however it may now mutate to a type of cell resistant to both types of therapy. In this model then the question becomes, how does one administer the various therapies so that the risk of total treatment failure (no more viable drugs) is minimized.

Seminal work was done in this field by Coldman and Goldie in several papers, e.g., Goldie and Coldman [43], Goldie et al. [44] and Coldman and Goldie [19]. We will focus on Coldman and Goldie [19], since it generalizes the previous works. It is assumed that there are *n* treatments available *T*_1_, …, *T*_*n*_, and 2^*n*^ different cell types present, each type specified by which subset of therapies the constituent cells are resistant to. Specifically, ${R}_{i_1,\dots,\ {i}_m}(t)$ is the number of cells at time *t* that are resistant to the therapies ${T}_{i_1}\dots,\ {T}_{i_m}$ and sensitive to all other therapies. The cell type *R*_0_ is sensitive to all therapies. In the absence of therapy it is assumed that all cells behave according to a pure birth process with birth rate *λ* per cell. During cell division events, mutations may occur and cells can acquire resistance to new types of drugs. Chemotherapy is modeled as an instantaneous probabilistic reduction in population of all sensitive cells according to a log cell kill rule. The authors then derive formulas for the probability of evolution of cells resistant to therapies within a finite time horizon. Coldman and Goldie, consider the case of two therapies and three distinct resistant cells in depth. In particular, let *P*_12_(*t*) be the probability that no cells with resistance to both therapies evolve by time*t*. Under symmetry assumptions on the efficacy of the two therapies and the behavior of the two singly resistant mutants, Coldman and Goldie [19] establishes that alternating therapies maximizes*P*_12_(*t*). Day computationally investigated relaxation of the symmetry assumptions and found that some non-alternating schedules could outperform the alternating schedule in that scenario [24]. In particular Day proposed a ‘worst drug first’ rule, this rule was investigated in further depth by Katouli and Komarova who considered a wide range of possible cyclic therapies [62]. In later works Murray and Coldman [72] and Coldman and Murray [20] extended the original model of Coldman and Goldie [19] to allow for toxicity constraints on normal tissues, simultaneous administration of multiple drugs, and included the possibility of inter-patient heterogeneity. In [18], Chen et al. further investigated the effects of asymmetry in the efficacy of the two possible therapies, and derived general conditions for the identification of optimal drug administration sequences. One potential shortfall of the Goldie and Coldman model is that the tumor cell populations grow exponentially ignoring possible effects of resource depletion. Chapter 9 of the monograph Martin and Teo [69] develops a deterministic model that allows for logistic and Gompertz growth in the tumor population. In this model they have four types of cells and two therapies, the authors develop an algorithm that searches for the schedule of therapies that leads to the maximal time until treatment failure. Note that this algorithm only identifies local optima though.

Despite the large amount of work done in this field there are still significant challenges remaining. In particular, previous works have looked at optimal schedules with only a small number of resistant types and potential therapies. Going forward, an important extension will be to develop methodologies that allow for the optimization of administration schedules for larger number of therapies and a larger number of resistant types. Another possible extension is to study minimization of probability of resistance in more complex stochastic models. Until now the stochastic models have all had essentially exponential growth properties, which are known to be inconsistent with tumor growth curves. An exciting challenge for the future is to minimize resistance probabilities in stochastic models that include density dependence.

### Stochastic analysis

There has been a large volume of work on the stochastic modeling of cancer evolution, e.g., see the monographs Kimmel and Axelrod [63] and Durrett [29]. Given this large body of work we will focus on stochastic models for the evolution of resistance under therapy. In the works of Komarova and Wodarz [66] and Komarova [65], Komarova and Wodarz extended the model of Coldman and Goldie by replacing a pure birth process with cell kill events due to therapy with a multi-type binary branching process. Here the types are representative of the therapies that the cells are resistant, and cells mutate to give rise to daughter cells with new types of resistance. In [36], Foo and Michor also consider a multi-type binary branching process, but they allow for time inhomogeneous birth and death rates and then identify dosing schedules that minimize risk of resistance subject to toxicity constraints. A follow up work [37] generalized this model to allow for arbitrary concentration curves and incorporated pharmacokinetic effects. Fla et al. constructed a stochastic model for the evolution of normal blood stem cells, wild-type leukemic stem cells, and mutated drug resistant leukemic stem cells [33]. A novel feature of this model is that it was a stochastic model that incorporated competition. The authors derived the Fokker-Planck equations governing the probability mass functions of the stochastic model and analyzed the possible equilibrium of the system. In a series of works Foo and Leder [35, 38] studied a branching process model for the evolution of heterogeneous cancer population undergoing therapy. In particular denote the drug sensitive cell population at time *t* by *Z*_0_(*t*) and the drug resistant cell population by *Z*_1_(*t*), with *Z*_0_(0) = *n* and *Z*_1_(0) = 0. The sensitive cell population is modeled as a subcritical binary branching process, that produces resistant cells at rate *μ* and each resistant cell initiates a super-critical branching process with random net growth rate. In these works the properties of the cancer cell population is investigated at the ‘crossover-time’:

$$ \xi = \min \left\{t>0:\ {Z}_1(t)>{Z}_0(t)\right\}. $$

In particular, Foo and Leder [35] study the relationship between *ξ* and the extinction time of the sensitive cell process. While Foo et al. [38] study the diversity properties of the resistant cell population at the time *ξ*. There are several standard metrics for diversity of a population, e.g. the number of distinct species present, the Simpson’s Index (probability two randomly chosen cells are genomically identical), and Shannon’s Index (related to Shannon’s Entropy, see e.g. [22]). In Foo et al. [38] they consider all three of these diversity measures. Lastly the work of Foo et al. [39] establishes a central limit theorem for *ξ* in the limit as the initial population *Z*_0_(0) goes to infinity, and identifies the effect of the random fitness distribution on the large *n* behavior of the crossover time *ξ*.

There are lots of open problems remaining in the topic of stochastic models of cancer cells undergoing therapy. An interesting extension would be to investigate the treatment process when spatially explicit models (such as [95]) are used.

## Discussion

Viewing tumors as an evolving population of cells has proven to be a useful tool in the study of cancer. Anti-cancer therapy clearly has the potential to impact the evolutionary trajectory of the tumor cell population. The behavior of this evolution is extremely interesting in the context of diverse tumor cell populations. For example, one might expect that therapy will select for cells with therapy resistance, thus leaving a more difficult to treat tumor. In order to achieve the best possible therapeutic results it is thus seems necessary to create treatment strategies that take into account the diversity present within a tumor and the evolutionary changes the tumor might undergo during therapy.

There has clearly been a significant amount of work done in the field of cancer therapy optimization. However, there are still lots of exciting problems remaining to be investigated. For example, there are few theoretical results about the structure of optimal radiotherapy schedules when studying heterogeneous populations. In the chemotherapy setting there are no suitable optimization methods for dealing with large amounts of heterogeneity present, i.e., large numbers of distinct cell types. There are several interesting open problems in the stochastic modeling and optimization framework. In particular, more work needs to be done in this area that incorporates cellular competition.

Perhaps the biggest challenge in the field of designing optimal cancer therapies, is bringing these optimized therapeutic schedules into the clinic. While there have been successes in the laboratory setting, e.g., Leder et al. [67], Gao et al. [41], successes in a clinical setting are quite rare.

## Abbreviations

CSC, cancer stem cell; DCC, differentiated cancer cells; DLQ, dual-compartment linear-quadratic; DSB, double strand breaks; LQ, linear-quadratic; OAR, organs-at-risk; TCP, tumor control probability

## Reviewers’ comments

### Reviewer’s report 1 Thomas McDonald, Biostatistics and Computational Biology, Dana-Farber Cancer Institute

Reviewer comments:

**Summary:**

The review provides a general overview of modeling therapy of tumors. It separates into radiotherapy and chemotherapy discussing historical and more recent models of each and the impact of heterogeneity that affect tumor response. The authors do a good job discussing radiotherapy beginning with the Linear-Quadratic model before moving into the various extensions too account for the four ‘Rs’. The section on Chemotherapy covers a wide range of work from the Coldman and Goldie models up to modern methods used and include a discussion of the next necessary steps and issues to tackle. Ultimately, this review provides a useful recap of the work done in mathematical modeling of radiotherapy and chemotherapy.

**Reviewer recommendations to authors:**

Major: The main suggestion is to include a few more pictures of some of the processes mentioned. The radiotherapy models could be illustrated with curves and example plots of tumor response curves showing the impact of heterogeneity as modeled in some of the articles cited.

The second part on chemotherapy seems lacking in the detail that the radiotherapy section got, and it may deserve a little more time or mathematical description of the work. The focus of the work is clearly radiotherapy, but explaining some of the chemotherapy models in a little more depth or describing a quick background of branching processes and their use would make the review more complete. A more careful proofreading is necessary. There are minor grammatical errors scattered throughout. An incomplete list is given below.

The first section on radiotherapy may be separated into subsections since the authors jumped between models abruptly.

Author’s response: *Thank you for your careful reading of the manuscript and helpful suggestions, we have addressed these comments*.

### Reviewer’s report 2 David Axelrod, Rutgers University

Reviewer comments:

**Summary:** Recommendation status: Endorse publication as a Review. Reviewers report: Summary of some mathematical modeling to optimize radiotherapy and chemotherapy, with brief mention of open problems, but little indication of whether or not the modeling has had a clinical impact, and if not why not. Not comprehensive or original, although useful as an entrance to the literature.

Author’s response: *Thank you for your careful reading of the manuscript and helpful suggestions, we have addressed these comments.*
